# Case report: The impact of dissociated response of immunotherapy on the treatment strategy of advanced head and neck cancer

**DOI:** 10.3389/fimmu.2024.1460480

**Published:** 2024-10-15

**Authors:** Siqing Jiang, Xin Li, Jin Li

**Affiliations:** ^1^ Department of Comprehensive Chemotherapy/Head and Neck Cancer, Hunan Cancer Hospital, The Affiliated Cancer Hospital of Xiangya School of Medicine, Central South University, Changsha, Hunan, China; ^2^ Department of Pain Management and Anesthesiology, The Second Xiangya Hospital, Central South University, Changsha, Hunan, China

**Keywords:** dissociated response, immunotherapy, advanced head and neck cancer, treatment strategy, conversion therapy

## Abstract

Some special therapeutic responses may appear during immunotherapy, such as hyperprogression, pseudoprogression and so on. Dissociated response of immunotherapy has been clinically reported in recent years mainly in lung cancer and kidney cancer. Since there were poor prognosis and simple treatment of advanced head and neck cancer, the application of immunotherapy in head and neck cancer has risen in recent years. But the dissociated response of immunotherapy in head and neck cancer is rarely reported. We reported two series of cases of advanced head and neck cancer that showed dissociated response after immunotherapy, tumor progression was assessed by imaging methods such as PET-CT, enhanced CT and enhanced MR, and reviewed the literature related to dissociated response in immunotherapy. We propose that the dissociated response of immunotherapy may affect the treatment strategy of advanced head and neck cancer, but more clinical analyses and researches are needed to confirm it.

## Introduction

Squamous cell carcinoma of the head and neck (HNSCC) is the seventh most common malignancy worldwide, with approximately 64% of patients already locally advanced at the time of initial diagnosis (1, 2). With the increased use of immunotherapy in Patients with advanced head and neck cancer (3, 4), some special therapeutic responses of immunotherapy, such as hyperprogression (5), pseudoprogression (6), dissociated response(DR) and so on, deserve our attention. The dissociated response of immunotherapy in head and neck cancer has been rarely reported. We present two cases of recurrent and metastatic head and neck tumors with immunotherapeutic dissociated response in order to provide new insights for clinical decision-making.

## Cases reports

**Figure 1:**
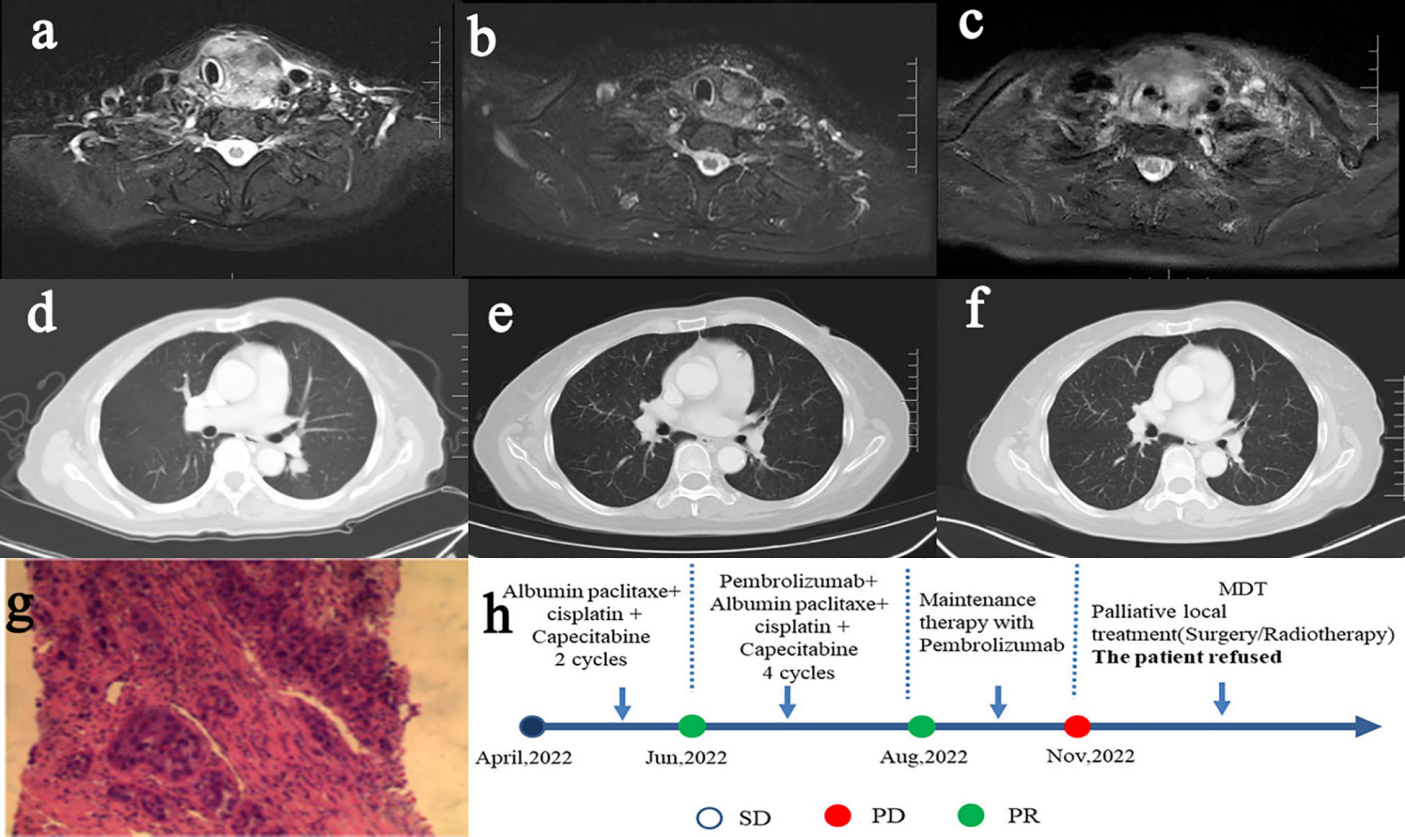
Contrast MR imaging of thyroid lesions (**A**, April 2022) (**B**, Aug 2022) (**C**, Nov 2022), contrast CT imaging of pulmonary metastases (**D**, April 2022) (**E**, Aug 2022) (**F**, Nov 2022), pathological HE staining **(G)**, treatment procedure and curative effect evaluation **(H)**.

Female patient in their 60s presented with a thyroid mass and lung metastases on imaging at the initial diagnosis (**Figures 1A, D**). The pathological report of the mass in left lobe of thyroid gland showed that it has been squamous cell carcinoma, and the immunohistochemical results demonstrated that malignant cells were positive for CK7 (regional positive), P40, CKpan, CK5/6, EMA, P16, PD-L1 (22C3) (CPS: > 50), EGFR, and negative for Calcitonin, CD117, CD5, CD56, CgA, CK20, Pax-8, SYN, Tg, NapsinA and TTF-1 immunohistochemical markers (**Figure 1G**). The patient was diagnosed with thyroid carcinoma (squamous cell carcinoma of the left lobe, cT2N1M1, stage IV lung metastases).

After completing 6 cycles of chemotherapy and 4 cycles of immunotherapy, the pulmonary metastases were evaluated as complete response (CR) (**Figures 1B, E**). In the immune maintenance stage, the primary lesions progressed but the metastatic lung lesions were still in CR after 3 cycles of immunotherapy (**Figures 1C, F**). After consultation of multi-disciplinary team (MDT), it was recommended to have local treatment of surgery or radiotherapy for thyroid lesions progress. However, the patient and her family refused further treatment of surgery or radiotherapy after consideration, and still asked for conservative treatment. Considering the persistent CR of pulmonary metastases and the willingness of the patient, we retained immunotherapeutic drugs, changed the chemotherapy regimen, and added targeted therapy. The treatment of the patient was shown as follows in **Figure 1H**.

**Figure 2:**
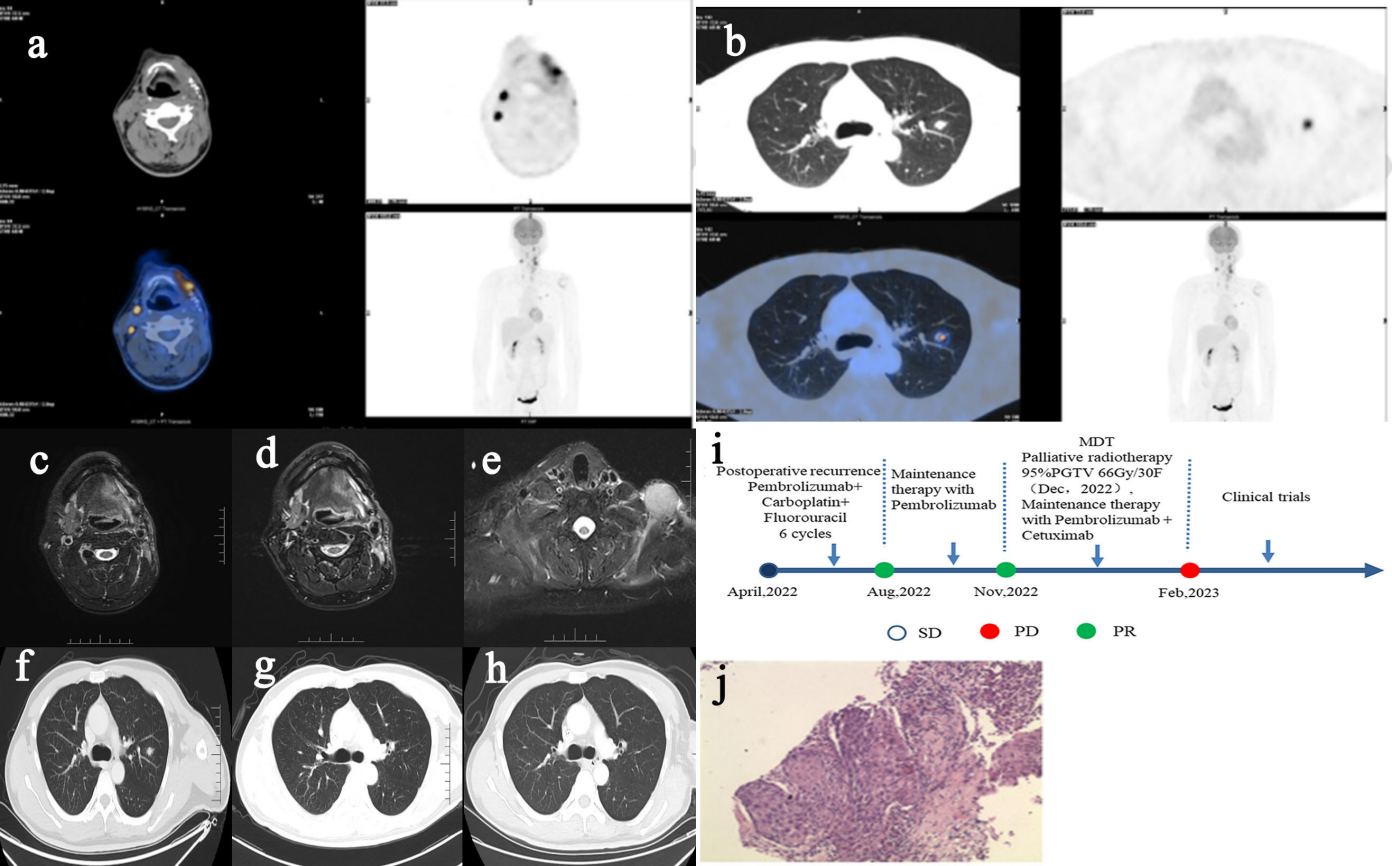
PET-CT imaging of recurrent lesions of head and neck (**A**, April 2022), PET-CT imaging of metastatic lesions of lung (**B**, April 2022), contrast MR imaging of recurrent lesions of head and neck (**C**, April 2022) (**D**, Nov2022), contrast MR imaging of new supraclavicular lesions that progressed after radiotherapy (**E**, Feb 2023), contrast CT imaging of metastatic lesions of lung (**F**, April 2022) (**G**, Nov2022) (**H**, Feb 2023), treatment procedure and curative effect evaluation **(I)**, pathological HE staining **(J)**.

Male patient in their 40s presented with postoperative recurrence of gingival carcinoma. Both PET-CT and contrast CT/MR of the patient suggested pulmonary metastases (**Figures 2A–C, F**). The pathology of the mass showed that it has been highly-moderately differentiated squamous cell carcinoma, and the immunohistochemistry demonstrated that malignant cells were positive for CK-pan, CK5/6, EGFR, Ki67 (35%), P16 (small focus positive), P40, p53, PD-L1 (22C3) (CPS:5) immunohistochemical markers (**Figure 2J**). The patient was diagnosed with postoperative recurrence of gingival carcinoma (moderately-well differentiated squamous cell carcinoma, rT2N2M1, stage IVc lung metastasis).

After 6 cycles of chemotherapy and immunotherapy, the primary lesions of head and neck was partial response (PR) and the metastatic lesions of lung was CR (**Figures 2D, G**). After consultation of MDT, the patient was advised to receive local radiotherapy. The patient developed new lesions at the time more than 1 month after radiotherapy, and progressive disease (PD) was considered, but the pulmonary metastases were still in the state of CR (**Figures 2E, H**). Then the patient was suggested to enter a clinical trial and he agreed. The treatment of the patient was shown as follows in **Figure 2I**. The above two patients had no special past history and no family history of malignancy.

## Discussion

At present, the dissociated response of immunotherapy is not well defined in radiological criteria guidelines. Generally speaking, it means that multiple target lesions have different degrees of therapeutic responses in the process of immunotherapy. According to the combination of different target lesions in clinic, Vaflard et al. have defined three types of DR (7): (1) one target lesion with CR/PR and one target lesion with PD (DR1); (2) one target lesion with stable disease (SD) and one target lesion with PD (DR2); (3) one target lesion with CR/PR and one target lesion with SD (DR3). According to the immune Response Evaluation Criteria in Solid Tumours (iRECIST), the overall response assessment of DR patients can be iPR, iSD, iUPD or iCPD. Then DR may be mistakenly classified as true progression by RECIST and iRECIST. Therefore, the occurrence of dissociated response may affect the decision of immunotherapy application. In addition, most current studies tend to believe that dissociated response in immunotherapy is an indicator with good prognosis (8–10), and prefer to retain immunotherapy after dissociated response occurs, which is different from the fact that dissociated response in chemotherapy and targeted therapy is an indicator of poor prognosis (11). The follow-up diagnosis and treatment program of the female patient we reported above referred to these studies and then retained immunotherapy. However, most of the cases in these published studies were lung cancer and other cancer types of solid tumors. When dissociated response appeared in head and neck cancer, the choice of immunotherapy was worthy of further discussion.

In the cases we provide, the distant metastases have achieved continuous CR during the treatment process, so whether the treatment of the primary lesion is still treated with systemic therapy or local treatment intervention (radiotherapy, surgery, etc.) can be considered is the focus of our next treatment strategy selection. It is noteworthy that Chinese researchers proposed localized nasopharyngeal radiotherapy for nasopharyngeal carcinoma patients with distant metastasis (non-liver metastasis) still had survival benefit (12). Therefore, based on the cases we provided, whether further refinement and stratification is needed to explore the differences of metastatic lesions with dissociated response may affect the decision of immunotherapy application, and then affect the prognosis and survival of patients.

The conversion therapy for colorectal cancer with liver metastasis has become a standard clinical practice. With the addition of immunotherapy, patients who had locally advanced head and neck cancer could achieve the goal of downstaging and benefit to reach the condition for focal treatment. At present, this clinical practice has been further confirmed by large-scale clinical trials (13).However, the studies on the conversion therapy for advanced head and neck cancer with distant metastasis were not sufficient. The cases reports we provided suggested that the dissociated response of immunotherapy in clinic had a significant impact on therapeutic strategies. Furthermore, because of the trailing effect of immunotherapy, some metastatic lesions continue subsided in the subsequent stage of treatment. Therefore, in the case of CR of distant metastatic lesions, whether there is survival benefit in continuing local treatment (surgery or radiotherapy) in head and neck lesions, further clinical studies are needed to confirm these results.

In the report of Bernard-Tessier A (9), a total of 360 patients with solid tumors were included, of which 24 cases of head and neck cancer had no dissociated response. But the researcher did not report the metastasis of the 24 cases of head and neck cancer in detail. The two cases we reported were both patients with lung metastases in head and neck cancer. According to the study of Vaflard P (7), if only focus on the organ where the target lesion is located and paying no attention to the type of primary tumor, the target lesion response rates and the probability of CR and PR were all higher in the lung and lymph node lesions. This is similar to CR of pulmonary lesions in the two patients of head and neck cancer with lung metastases we reported above. We speculate that the mechanism may be as follows: tumor heterogeneity, differences in the PD-L1 expression (14), and differences in the immune microenvironment in different organs (15).

In addition, PET/CT was used in the diagnosis of distant metastases in one of the HNSCC cases we offered. PET/CT is involved in the diagnosis and treatment and management of HNSCC, and plays an important role in the comprehensive evaluation before treatment, the determination of the target area of surgery and radiotherapy, the identification of *in situ* recurrence and inflammation, the identification of distant metastatic lesions, the evaluation of treatment efficacy, and prognostic follow-up (16, 17). PET/CT has been shown to improve the accuracy of lymph node staging on MRI of the neck in untreated laryngeal cancer and to increase the prognosis of survival outcomes through the use of multiple PET measures (18). With the comprehensive application of immunotherapy in tumor treatment, the development of immunotherapy imaging for cancer provides an important reference for clinical decision-making, especially for the identification of hyperprogression, pseudoprogression, and dissociative response (19).

In conclusion, we provide two cases reports of head and neck cancer with dissociated response of immunotherapy, which puts forward new ideas and challenges for the conversion therapy of immunotherapy in advanced head and neck cancer.

## Data Availability

The original contributions presented in the study are included in the article/supplementary materials, further inquiries can be directed to the corresponding author/s.

## References

[1] Vos JL Elbers J Krijgsman O Traets J Qiao X van der Leun AM . Neoadjuvant immunotherapy with nivolumab and ipilimumab induces major pathological responses in patients with head and neck squamous cell carcinoma. Nat Commun. (2021) 12:7348. doi: 10.1038/s41467-021-26472-9 34937871 PMC8695578

[2] Qiu H Cao S Xu R . Cancer incidence, mortality, and burden in China: a time-trend analysis and comparison with the United States and United Kingdom based on the global epidemiological data released in 2020. Cancer Commun (Lond). (2021) 41:1037–48. doi: 10.1002/cac2.12197 PMC8504144 34288593

[3] Harrington KJ Ferris RL Blumenschein GJ Colevas AD Fayette J Licitra L . Nivolumab versus standard, single-agent therapy of investigator’s choice in recurrent or metastatic squamous cell carcinoma of the head and neck (CheckMate 141): health-related quality-of-life results from a randomised, phase 3 trial. Lancet Oncol. (2017) 18:1104–15. doi: 10.1016/S1470-2045(17)30421-7 PMC6461049 28651929

[4] Burtness B Harrington KJ Greil R Soulieres D Tahara M de Castro GJ . Pembrolizumab alone or with chemotherapy versus cetuximab with chemotherapy for recurrent or metastatic squamous cell carcinoma of the head and neck (KEYNOTE-048): a randomised, open-label, phase 3 study. Lancet. (2019) 394:1915–28. doi: 10.1016/S0140-6736(19)32591-7 31679945

[5] Champiat S Dercle L Ammari S Massard C Hollebecque A Postel-Vinay S . Hyperprogressive disease is a new pattern of progression in cancer patients treated by anti-PD-1/PD-L1. Clin Cancer Res. (2017) 23:1920–8. doi: 10.1158/1078-0432.CCR-16-1741 27827313

[6] Seiwert TY Burtness B Mehra R Weiss J Berger R Eder JP . Safety and clinical activity of pembrolizumab for treatment of recurrent or metastatic squamous cell carcinoma of the head and neck (KEYNOTE-012): an open-label, multicentre, phase 1b trial. Lancet Oncol. (2016) 17:956–65. doi: 10.1016/S1470-2045(16)30066-3 27247226

[7] Vaflard P Paoletti X Servois V Tresca P Pons-Tostivint E Sablin MP . Dissociated responses in patients with metastatic solid tumors treated with immunotherapy. Drugs R D. (2021) 21:399–406. doi: 10.1007/s40268-021-00362-3 34562258 PMC8602606

[8] Tozuka T Kitazono S Sakamoto H Yoshida H Amino Y Uematsu S . Dissociated responses at initial computed tomography evaluation is a good prognostic factor in non-small cell lung cancer patients treated with anti-programmed cell death-1/ligand 1 inhibitors. BMC Cancer. (2020) 20:207. doi: 10.1186/s12885-020-6704-z 32164651 PMC7066771

[9] Bernard-Tessier A Baldini C Castanon E Martin P Champiat S Hollebecque A . Patterns of progression in patients treated for immuno-oncology antibodies combination. Cancer Immunol Immunother. (2021) 70:221–32. doi: 10.1007/s00262-020-02647-z PMC10973750 32700090

[10] Sato Y Morimoto T Hara S Nagata K Hosoya K Nakagawa A . Dissociated response and clinical benefit in patients treated with nivolumab monotherapy. Invest New Drugs. (2021) 39:1170–8. doi: 10.1007/s10637-021-01077-7 33566254

[11] Dong ZY Zhai HR Hou QY Su J Liu SY Yan HH . Mixed responses to systemic therapy revealed potential genetic heterogeneity and poor survival in patients with non-small cell lung cancer. Oncologist. (2017) 22:61–9. doi: 10.1634/theoncologist.2016-0150 PMC5313275 28126915

[12] You R Liu YP Huang PY Zou X Sun R He YX . Efficacy and safety of locoregional radiotherapy with chemotherapy vs chemotherapy alone in *de novo* metastatic nasopharyngeal carcinoma: A multicenter phase 3 randomized clinical trial. JAMA Oncol. (2020) 6:1345–52. doi: 10.1001/jamaoncol.2020.1808 PMC7378870 32701129

[13] Ferris RL Spanos WC Leidner R Goncalves A Martens UM Kyi C . Neoadjuvant nivolumab for patients with resectable HPV-positive and HPV-negative squamous cell carcinomas of the head and neck in the CheckMate 358 trial. J Immunother Cancer. (2021) 9(6). doi: 10.1136/jitc-2021-002568 PMC8183204 34083421

[14] Moutafi MK Tao W Huang R Haberberger J Alexander B Ramkissoon S . Comparison of programmed death-ligand 1 protein expression between primary and metastatic lesions in patients with lung cancer. J Immunother Cancer. (2021) 9(4). doi: 10.1136/jitc-2020-002230 PMC8039214 33833050

[15] Oliver AJ Lau P Unsworth AS Loi S Darcy PK Kershaw MH . Tissue-dependent tumor microenvironments and their impact on immunotherapy responses. Front Immunol. (2018) 9:70. doi: 10.3389/fimmu.2018.00070 29445373 PMC5797771

[16] Cammaroto G Quartuccio N Sindoni A Di Mauro F Caobelli F . The role of PET/CT in the management of patients affected by head and neck tumors: a review of the literature. Eur Arch Otorhinolaryngol. (2016) 273:1961–73. doi: 10.1007/s00405-015-3651-4 25971995

[17] Caldarella C De Risi M Massaccesi M Micciche F Bussu F Galli J . Role of (18)F-FDG PET/CT in head and neck squamous cell carcinoma: current evidence and innovative applications. Cancers (Basel). (2024) 16(10). doi: 10.3390/cancers16101905 PMC11119768 38791983

[18] Al-Ibraheem A Abdlkadir AS Al-Adhami D Hejleh TA Mansour A Mohamad I . The prognostic and diagnostic value of [(18)F]FDG PET/CT in untreated laryngeal carcinoma. J Clin Med. (2023) 12(10). doi: 10.3390/jcm12103514 PMC10218884 37240619

[19] Dercle L Sun S Seban RD Mekki A Sun R . Emerging and evolving concepts in cancer immunotherapy imaging. Radiology. (2023) 306:32–46. doi: 10.1148/radiol.210518 36472538

